# Dynamic Responses of Blast-Loaded Shallow Buried Concrete Arches Strengthened with BFRP Bars

**DOI:** 10.3390/ma15020535

**Published:** 2022-01-11

**Authors:** Jianqin Wu, Jiannan Zhou, Ying Xu, Xinli Kong, Peng Wang, Bo Wang, Chengjie Zhao, Fengnian Jin, Wenye Wang, Fengxia Wang

**Affiliations:** State Key Laboratory of Disaster Prevention & Mitigation of Explosion & Impact, Army Engineering University of PLA, Nanjing 210007, China; lw2007985@gmail.com (J.W.); zjn_0414@163.com (J.Z.); wbo8010@gmail.com (B.W.); zhaochengjie7230@163.com (C.Z.); jinfn2020@163.com (F.J.); wangwenye9012@gmail.com (W.W.); qdsftawfx@163.com (F.W.)

**Keywords:** concrete arch, BFRP bars, blast resistance, damage

## Abstract

This paper proposes a prefabricated basalt fiber reinforced polymer (BFRP) bars reinforcement of a concrete arch structure with superior performance in the field of protection engineering. To study the anti-blast performance of the shallow-buried BFRP bars concrete arch (BBCA), a multi-parameter comparative analysis was conducted employing the LS-DYNA numerical method, which was verified by the results of the field explosion experiments. By analyzing the pressure, displacement, acceleration of the arch, and the strain of the BFRP bars, the dynamic response of the arch was obtained. This study showed that BFRP bars could significantly optimize the dynamic responses of blast-loaded concrete arches. The damage of exploded BBCA was divided into five levels: no damage, slight damage, obvious damage, severe damage, and collapse. BFRP bars could effectively mitigate the degree of damage of shallow-buried underground protective arch structures under the explosive loads. According to the research results, it was feasible for BFRP bars to be used in the construction of shallow buried concrete protective arch structures, especially in the coastal environments.

## 1. Introduction

As important structural members, reinforced concrete arches are widely used in underground protective structures [1,2,3]. With the development of defense engineering, the protective structure is required not only to have excellent mechanical properties but also to be used in some special environments, such as under explosion. Besides, there are higher requirements for the durability of protective engineering. Therefore, it is necessary to take scientific and reasonable measures to enhance the blast-resistance performance and the durability of traditional structures.

FRP (fiber reinforced polymer) materials have good corrosion resistance, lightweight, high strength, and excellent magnetic transparency. Hence, they have great potential for improving the overall performance of concrete structures [4,5,6,7,8]. Common FRP materials include BFRP (basalt fiber reinforced polymer), GFRP (glass fiber reinforced polymer), AFRP (aramid fiber reinforced polymer), and CFRP (carbon fiber reinforced polymer). Among them, BFRP has the characteristics of more stable physical and chemical properties and higher economic performance. In civil engineering applications, BFRP materials are often made into bars to replace steel bars in reinforced concrete structures. [9,10,11,12].

The elastic modulus of BFRP bars is low and there is no yield phase before the bar breaks. There has been extensive research to systematically study the tensile strength, the creep resistance, the impact resistance, and other mechanical properties of BFRP bars. Studies have included replacement of steel bars with BFRP bars, to discover their effect on the overall performance of concrete members [13,14]. In the field of civil construction, FRP bars are commonly used to enhance the durability and the strength of structures to meet certain specific functions during usage [15]. Many studies have also proved that the overall mechanical performances of concrete components using BFRP bars is greatly improved [16]. Wu et al. [17,18] found that BFRP could effectively improve the seismic performance of concrete columns.

BFRP bars are not only used in beams, slabs, columns, and other common components [19,20,21] but are also used in the construction of concrete arch structures [22]. Tang et al. [23,24] used BFRP bars instead of steel bars to make concrete arch members and found that the concrete arch with BFRP bars had comparable resistance to the concrete arch with steel bars, under air-explosion. Zhao et al. [25] found that the BFRP-steel composite bars reinforced concrete arches exhibited a higher load-bearing capacity and more safety redundancy than the steel bars reinforced concrete arches. Through blast-resistance experimental research, they found that using BFRP bars instead of steel bars could enable concrete arches to perform better in protective structures.

Explosion experiments are usually very complicated and costly. Therefore, it is difficult to conduct large-scale experiments repeatedly. However, numerical simulation can better reveal the dynamic responses of structures under the action of explosive loads by accurately controlling each explosion variable [26]. In previous studies, less attention has been paid to the dynamic response of BFRP reinforcement members under explosive loads. For common structures in engineering [27], some researchers conducted limited anti-explosion experiments, but they could not well summarize the dynamic response laws of BFRP reinforced concrete arches. 

In this paper, BFRP bars were selected as the reinforcement material to strengthen the concrete arch structure, and various simulation conditions were designed to investigate the influences of multiple factors. By combining the experimental and the numerical simulation results, a more comprehensive analysis was conducted to study the dynamic responses of shallow-buried concrete arches under explosive loads. These works provide an economical and reasonable design basis for improving the anti-blast performance of arch structures in protective engineering.

## 2. Finite Element Model

### 2.1. Model Parameters

**Figure 1:**
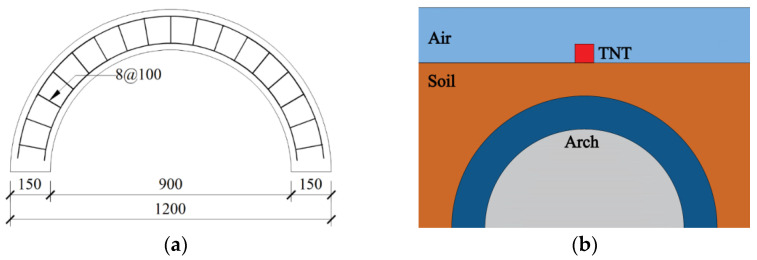
Schematic diagram of the BBCA component: (**a**) parameters of the BFRP reinforcement cage (mm), and (**b**) shallow buried protective concrete arch.

Figure 1a shows the model parameters of the BFRP bars concrete arch (referred to as BBCA) with an inner diameter of 900 mm, an outer diameter of 1200 mm, a thickness of 150 mm, and a transverse width of 300 mm. The longitudinal bars and the transverse stirrups were all BFRP bars. The schematic diagram of the blast-loaded shallow buried concrete arch is shown in Figure 1b.

### 2.2. Unit Division

**Figure 2:**
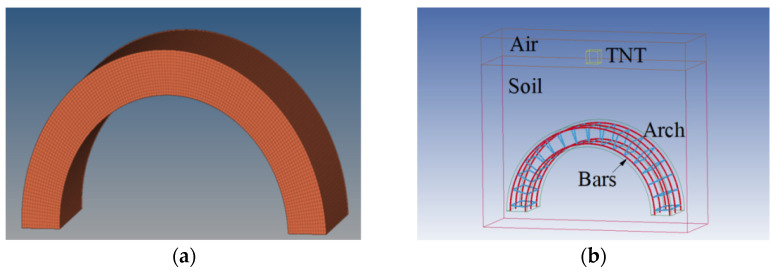
The meshing of (**a**) concrete arch structure, and (**b**) BFRP bars.

The HyperMesh software was used to establish a high-precision model. For the concrete arch structure, the C3D8R hexahedral reduced integral element was used for meshing as shown in Figure 2a. The element size was controlled in the range of 10–15 mm. There were 150 elements in the arch ring direction, 30 elements in the thickness direction, and 15 elements in the width direction, with 67,500 elements in total. For the BFRP bars (Figure 2b), BEAM elements were used for meshing, with 4034 elements in total.

### 2.3. Material Model Settings

The model consists of five material parts: air, TNT, soil, concrete, and BFRP bars [28]. The 008# constitutive model (*MAT_HIGH_EXPLOSIVE_BURN) was adopted for the explosive material. According to the JWL equation, the pressure generated by the explosion can be expressed as

(1) $$p=p\left(V,E\right)=A\left(1-\frac{\omega }{R1V}\right)e^{-R1V}+B\left(1-\frac{\omega }{R2V}\right)e^{-R2V}+\frac{\omega E}{V}$$

where *A*, *B*, *R*1, *R*2, and *ω* are explosive specific constants; the last term on the right-hand side is a low pressure (large expansion) term; *V* is the ratio of the volume of explosive products to the volume of unexploded explosives, and *E* is the detonation energy per unit volume.

Therefore, *E* has the unit of Pa. *A*, *B*, *R*1, *R*2, ω, and *V* are dimensionless parameters. It is recommended to use the unit system of the gram, centimeter, and microsecond when a model includes high explosives. The remaining parameters involved are shown in Table 1.

Air was simulated by the 009# constitutive model (*Mat_Null) and the EOS_LINEAR_POLYNOMIAL state equation. The specific values of the involved parameters are shown in Table 2.

This material model was used to simulate air. Hence, the parameters of PC, MU, TEROD, and CEROD were zero. C1, C2, C3...C9 were the 0, 1, 2, 3...9th polynomial equation coefficients which were zero when the state equation was used to simulate air.

The 005# constitutive model (*MAT_SOIL_AND_FOAM_TITLE) was adopted for the soil material. The material parameters are shown in Table 3.

The 159# constitutive model (*MAT_CSCM_CONCRETE_TITLE) was adopted for the concrete material, which could effectively simulate the mechanical properties of the concrete materials under dynamic loads such as blast and impact. The material parameters are shown in Table 4.

The 003# constitutive model (*MAT_PLASTIC_KINEMATIC_TITLE) was adopted for the BFRP material, which could effectively simulate the anisotropic mechanical properties of composite materials. The material parameters are shown in Table 5.

### 2.4. Explosion Setting

The process of the TNT explosion acting on the concrete composite arch structure was a process with fluid-solid coupling. Therefore, the fluid-solid coupling analysis was adopted between the explosive air and the concrete arch structure. To achieve fluid-solid coupling, the concrete composite arch structure was established as a Lagrangian element and placed in the Euler grid of the air. The explosive was also in the Euler grid to realize the flow in the air after the explosion. The interface between the air area and the Lagrangian area realized the continuity of contact and displacement through a coupling algorithm. A rigid bottom plate was set under the arch feet to restrain the longitudinal displacement of the arch, which can prevent the arch model from detaching from the bottom plate during the explosion. The damage of the concrete elements is set as eroded when the damage exceeds 0.99 and the maximum principal strain exceeds 1.

Since the slip between the BFRP bars and the concrete was not considered in this study, the common node connection mode was adopted between the BFRP bars and the concrete. The numerical simulation software was used to calculate multiple working conditions. There were 18 working conditions, as shown in Table 6. They were arranged and numbered according to the scaled distance; the scaled distance is used to characterize the magnitude of the explosion effect, and is the ratio of the standoff to the one-third power of the explosion mass.

## 3. Verification of the Numerical Method

### 3.1. Experimental Research

To investigate the dynamic response of the BBCA under the blast load and verify the reliability of the model, the simulation results were compared with the explosion experimental results. An arched concrete member made of 8 mm BFRP bars with an arch height of 600 mm was utilized in these experiments and it was buried in the soil. The dynamic response of the arch structure was studied under the explosion of 0.2 kg TNT explosives with a standoff distance of 0.15 m.

**Figure 3:**
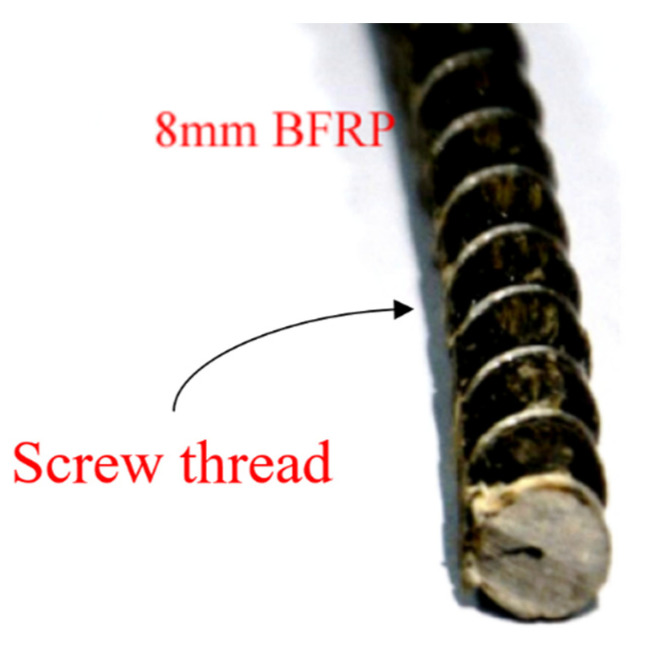
The BFRP bar with screw thread.

BFRP bars are composed of basalt fiber and resin matrices. In particular, the process is derived from the traditional wet layup method, but it is automated from tube to bar in one stage. The bars are wrapped during the process with a helix to give the desired screw thread form to achieve superior bond strength with concrete. As shown in Figure 3, the BFRP bars were provided by Shandong Safety Industrial Company, and the related material properties of reinforcement samples are listed in Table 7, which were directly acquired from the manufacturer, Shandong Safety Industrial Company.

The longitudinal bars of the concrete arch were arranged as 3 upper and lower bars. The spacing of the stirrups was 100 mm, the longitudinal bars and the stirrup were both 8 mm BFRP bars, and the bar ratio was 0.447%. The inner diameter of the arch was 900 mm, the outer diameter was 1200 mm, the thickness was 150 mm, and the lateral width was 300 mm.

**Figure 4:**
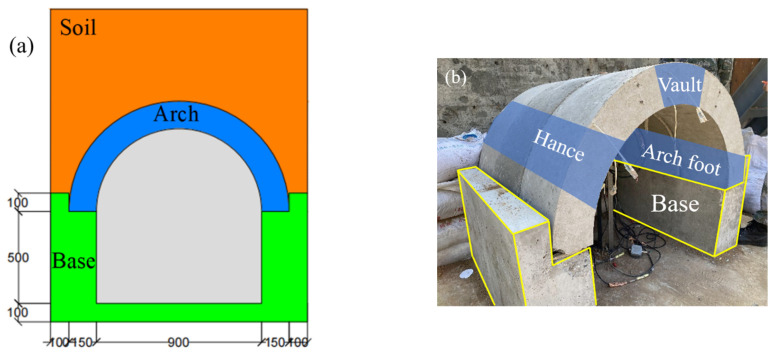
(**a**) Schematic diagram of the shallow buried BBCA (mm), and (**b**) Assembling of the experimental arches and the base [25].

During the experiments, the arch structure was shallowly buried in the soil. As shown in Figure 4, after the arch members were assembled into a whole structure on the base, soil bags and concrete slabs were piled around the base to block out the overburdened area, then the soil was filled in and the structure was buried. It is worth noting that the shape of the support is L-shaped: when the arch structure is placed on the support, there is a constraint on its horizontal displacement, and because the arch structure is in a buried state, the soil will also restrict the movement of the arch structure. In the theoretical analysis, the arch can be simplified into a two-hinged arch structure.

**Figure 5:**
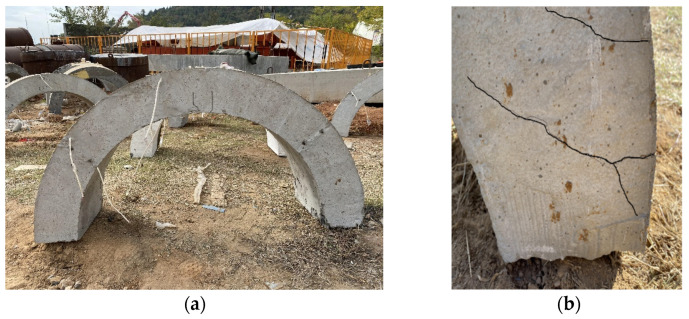
Macroscopic damage to the blast-loaded arches: (**a**) two cracks of the inner side of the vault, and (**b**) shear cracks at arch foot.

As shown in Figure 5, after the explosion, there were two vertical bending cracks on the lateral side of the vault. One of the cracks penetrated underneath the arch vault and the damage was slight. Also, diagonal shear cracks could be found at the arch foot.

### 3.2. Comparison and Validation

**Figure 6:**
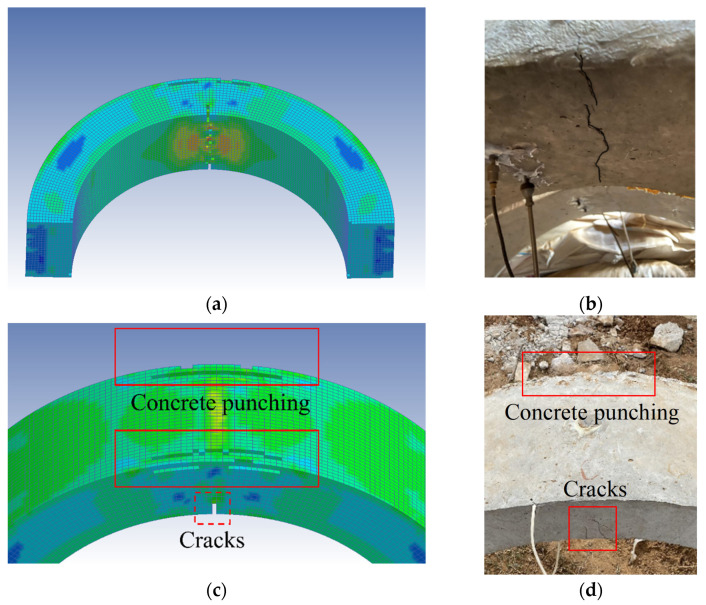
The penetrating concrete cracks of inner sides of (**a**) the simulated and (**b**) the experimental arch, the failures on the lateral sides of the vault of (**c**) the simulated and (**d**) the experimental arch.

The LS-DYNA software was used to conduct the numerical simulation to calculate pressure, displacement, and acceleration at the vault of the BBCA structure. Figure 6 shows the macroscopic failures of the simulated arch under the denotation of 0.2 kg TNT with a standoff distance of 0.15 m.

Under the blast condition of 0.15 m and usage of 0.2 kg TNT, many concrete elements were destroyed at the vault of the simulated arch. There were cracks inside of the arch and the sides of the arch were damaged. An oblique circular crack was produced at the arch foot. Based on the crack development and the spalling of the concrete, the simulated and experimental damages were close.

**Figure 7:**
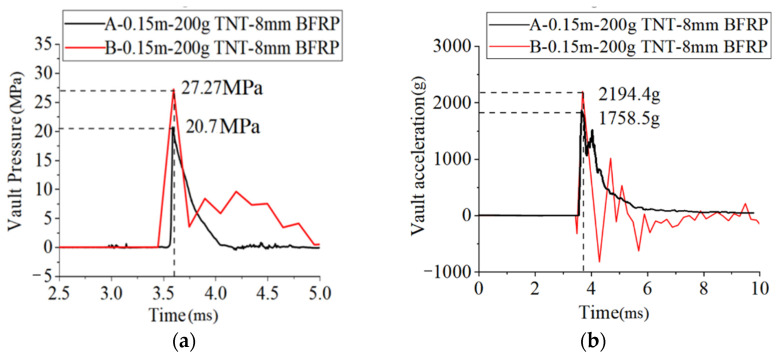
Comparison of the experimental and simulated time history curves of (**a**) the dynamic pressure, and (**b**) the acceleration at the vault. (Curve A: experimental result, curve B: simulation result).

Under the same working conditions, the experimental and simulation results were analyzed. Figure 7 displays the comparison of the dynamic pressures and accelerations at the vault.

In Table 8, the peak pressure and acceleration values of the experiments and the simulation are compared. Under the action of the explosive load, the peak pressure and acceleration of the vault in the experiments reached 20.7 MPa and 1758.5 g, respectively, while those in the simulation reached 27.3 MPa and 2197.4 g, respectively. As shown in Figure 6, the pressure and acceleration curves of the experiments and the simulation were close, and the peak values differed by 24% and 20%, respectively. Considering the problem of the compactness of the covering soil during the experiments, the errors were within a reasonable range. Through a series of comparisons, it was proved that the numerical model and the simulation method in this paper were reliable.

## 4. Simulation Results and Discussion

In the modeling method, 18 kinds of working conditions for the explosion simulation were given according to the scaled distance. The numerical simulation calculation was carried out and the calculation results were analyzed and discussed.

### 4.1. Macroscopic Damage Analysis

**Figure 8:**
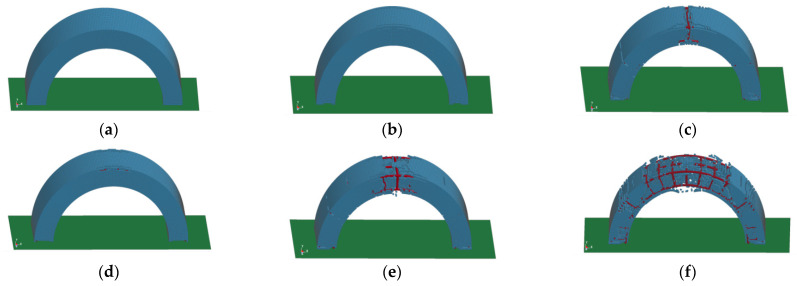
Macroscopic damages under various working conditions. (**a**) 0.5 kg TNT, 0.4 m; (**b**) 1 kg TNT, 0.4 m; (**c**) 3 kg TNT, 0.4 m; (**d**) 0.2 kg TNT, 0.15 m; (**e**) 0.5 kg TNT, 0.15 m; (**f**) 1 kg TNT, 0.15 m.

The BBCAs were damaged to various degrees under different explosion loads. When the standoff distance was 0.4 m and the amount of TNT was small, such as 0.5 kg or 1 kg, the arch structure almost maintained elastic deformation without significant damage, while there was only slight damage on both sides of the vault, categorized as ‘no damage’ or ‘slight damage’. When the amount of TNT was increased to 3 kg, the arch structure was significantly damaged. Many concrete units at the vault were broken and there was a through crack around the vault, leaving the BFRP bars exposed. There were also large cracks inside the vault. The cracks were divergent and there were many cracks and breaks on the hance. In addition, there were obvious cracks that penetrated the concrete arch outside of the hance. Oblique cracks appeared on the arch foot and the damage was severe. When the standoff distance was 0.15 m, the macroscopic damage of the arch structure became more and more serious with an increase in the amount of TNT. When the amount of TNT was 0.2 kg, as shown in Figure 8a,b, only the concrete on both sides of the vault was damaged, and the damage was slight. However, when the amount of TNT was 0.5 kg and 1 kg, the arch structure showed concrete fragmentation in a large area and the BFRP bars were directly exposed. The failure developed starting from the vault and gradually extended along the stress path of the vault, the hance, and the arch foot. When the amount of TNT was 1 kg, the arch structure collapsed and failed to work.

The development of concrete cracks had a certain pattern. Under the action of the explosion load, the structural cracks first started from both sides of the vault because of the diffraction phenomenon of the blast shock wave. When the shock wave reached the center of the vault for the first time, it was blocked and bypassed the center to both sides; the structure had no damage or slight damage. With an increase in the explosion effect, the concrete on both sides was completely broken; the cracks spread vertically in the center of the vault and then a ring-shaped crack penetrated the vault. Due to the pit-forming effect of the explosion, there was a depression in the center of the vault exposing the internal bars. The concrete on the inner side of the vault was stretched exceeding the ultimate strain of the concrete, and the inner concrete was peeled off. At this time, the arch structure changed from obvious damage to severe damage. With a continued increase in the magnitude of the explosion, the vault had obvious displacement, the curvature of the arch structure was changed, the plastic hinge formed at the vault, and the stress of the structure redistributed. The shear force at the hance increased and the transverse cracks gradually developed into circular cracks. Because the arch foot was restrained by hinges, diagonal cracks gradually developed, and a part of the concrete was crushed. When the explosion load exceeded the ultimate bearing capacity of the arch structure, the concrete peeled off and the arch structure collapsed.

From Figure 8a–f, we can see that these figures could be sorted according to the scaled distance. It is found that with the same standoff distance, the smaller the scaled distance was, the more serious was the damage to the structure. However, when the standoff distance was different, the damage was not so serious even with a small-scaled distance. The scaled distance in Figure 8c was smaller than that in Figure 8d, but the damage in Figure 8c was more serious than that in Figure 8d. By comparing the damage effects, it is found that the standoff distance is an important factor with the same amount of TNT. Comparing Figure 8a–c,f, when the standoff distance was 0.4 m, the arch structure did not have obvious damage, and it could still maintain structural integrity. However, when the standoff distance was 0.15 m, the arch structure was seriously damaged, and the strain of the BFRP bars reached a high value. Therefore, the standoff distance should be the main factor to be considered in anti-explosion design.

### 4.2. Pressure at the Vault

There were many working conditions in this study. To facilitate the analysis, some working conditions were taken for the analysis, their laws were summarized, and then the whole set of working conditions was studied. When the standoff distance was 0.4 m and the amount of TNT was 0.5 kg, the shapes of pressure curves of the BBCA vaults with diameters of 8 mm, 12 mm, and 16 mm were almost the same, and the peak pressure reached 3.05 MPa. At the end of the explosion, the pressure on the vault was zero and there was no residual stress.

**Figure 9:**
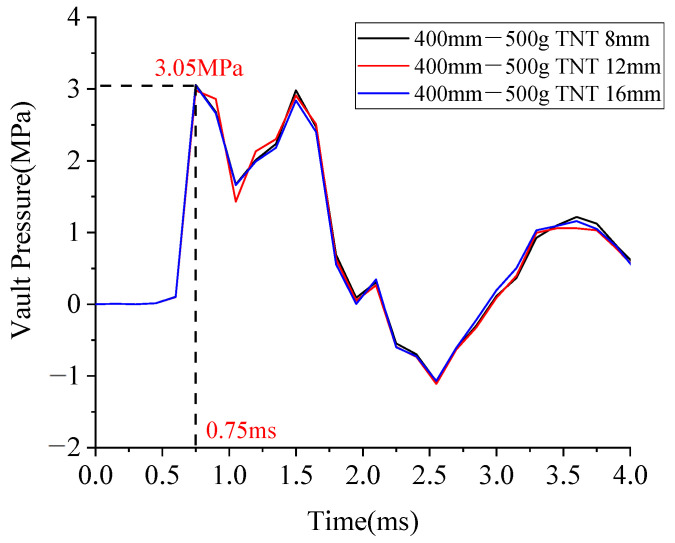
Pressure on the arch vault under a standoff distance of 0.4 m and an amount of TNT of 0.5 kg.

It can be seen from Figure 9 that the diameter of the BFRP bars had no significant effect on the pressure on the vault. Therefore, the time history curves of the pressure of the BFRP bars with a diameter of 8 mm were used for the comparative analysis.

**Figure 10:**
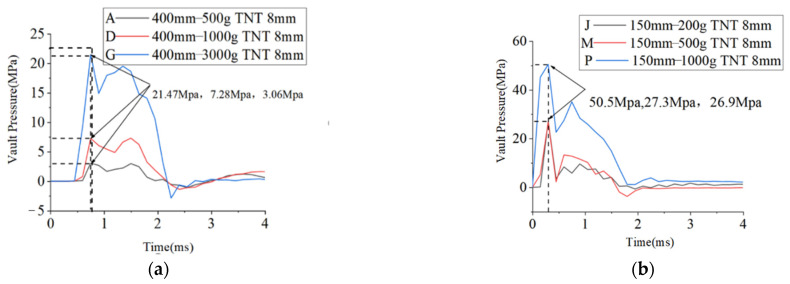
Dynamic pressure at arch vault under the conditions of (**a**) A, D, and G, and (**b**) J, M, and P.

Figure 10 shows that the dynamic pressure at the vault under the conditions of A, D, G, J, M, and P. The calculation formula of the peak value of the free field pressure in the soil under the explosion is given as follows [29]:

(2) $$P_{0}=f\rho c\cdot 160\cdot {\left(\bar{R}\right)}^{-n}$$

where f is the coupling coefficient of the soil and the air, and 0.14 can be taken for the explosive touchdown explosion corresponding to this condition; ρ is the density of the soil mass, c is the wave velocity in the soil, *n* is the attenuation coefficient of the explosion wave, which is normally 2.75, and $\left(\bar{R}\right)$ is the scaled distance that can be calculated according to the explosive and the standoff distance.

By replacing the values of various parameters in Equation (1) with the SI units, the calculation formula of the peak value (MPa) of the free field pressure in the soil can be obtained:

(3) $$P_{0}=3.836\times 10^{-6}\cdot f\rho c{\left(\bar{R}\right)}^{-n}$$

Using the scaled distance of each group to calculate P_1_, the pressure on the vault of each group was recorded and the Table 9 was obtained.

**Figure 11:**
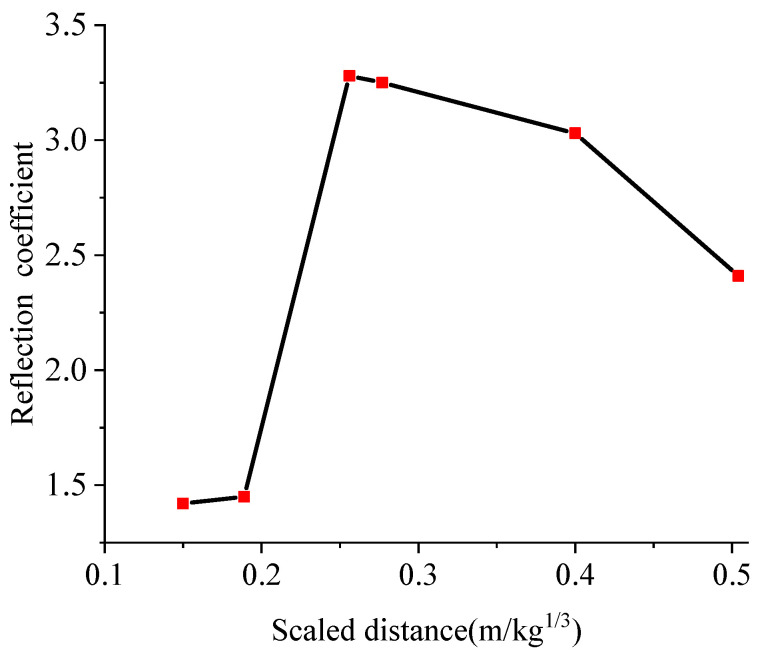
Relationship between the scaled distance and the reflection coefficient.

In Table 9, *P*_0_ is the calculated value of the pressure at the vault, and *P*_1_ is the simulated value of the pressure at the vault. The value of *P*_0_*/P*_1_ is the reflection coefficient of the explosion load in the soil and the arch structure. Under the working conditions of A, D, and G, that is, when the standoff distance was 0.4 m and the amount of TNT was 0.5 kg, 1 kg, and 3 kg, respectively, the reflection coefficient was inversely proportional to the scaled distance. However, under the working conditions of J, M, and P, the reflection coefficient was proportional to the scaled distance. Figure 11 shows the relationship between the scaled distance and the reflection coefficient. The largest reflection coefficient was observed at the scaled distance of 0.3 m/kg^⅓^.

By calculating the scaled distance, the peak pressure of the vault can be effectively predicted, so the structure could be designed for achieving the targeted explosion resistance. Under the impact of explosion, the pressure often reaches its peak point in a short time. Thus, it was meaningful to study the effect of the working conditions. Table 6 lists the impulses under the working conditions of A, D, G, J, M, and P.

Combining Table 10 and Figure 10, it could be found that, with the same standoff distance, the impulse increased with increasing the amount of TNT. With the same amount of TNT, the smaller was the standoff distance, the greater was the impulse, and the shorter was the time to reach the peak pressure.

### 4.3. Displacement of the Vault

**Figure 12:**
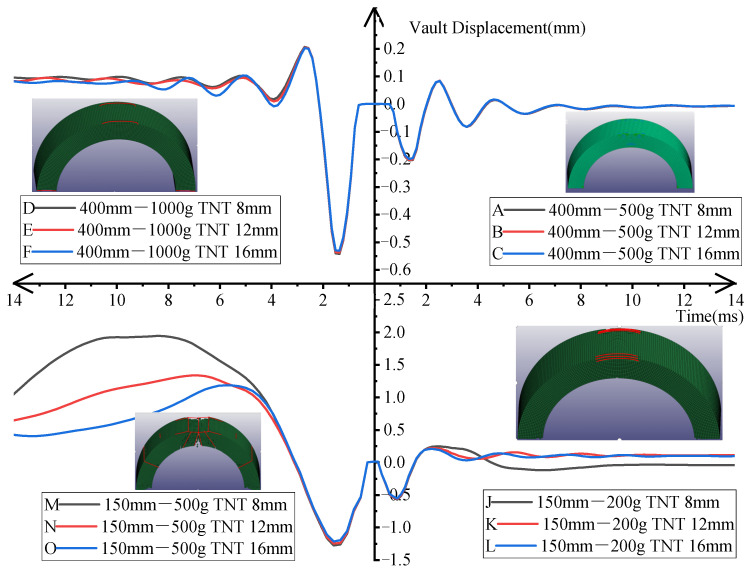
Time history curves of the dynamic displacement at the vault under various conditions.

When simulating the effect of the explosion, the arch structure was destroyed with a small standoff distance and the displacement of the concrete unit at the vault ‘blew up’. Therefore, the dynamic displacement curves with a standoff distance of 0.4 m and an amount of TNT of 0.5 kg and 1 kg as well as with a standoff distance of 0.15 m and an amount of TNT of 0.2 kg and 0.5 kg were taken for the comparative study. The results are shown in Figure 12.

When the standoff distance was 0.4 m and the amount of TNT was 0.5 kg, the displacement curves of the three BBCAs were almost the same. The first displacement peak point appeared immediately after the explosion and then the second peak point appeared due to the rebound. However, the second peak value was less than the first peak value. After that, the displacement of the arch fluctuated sinusoidally until the displacement returned to zero, indicating that the arch structure was still in the elastic stage and could maintain its original shape. When the amount of TNT increased to 1 kg, the displacement peaks of the three BBCAs were still almost the same, but there were slight differences in the elastic recovery stage. At this time, the arch structures were only slightly damaged on both sides of the vault. When the standoff distance was 0.15 m and the amount of TNT was 0.2 kg, the BBCAs with three diameters showed different responses after the arch structure reached the peak displacement. The 8 mm diameter BBCA slowly descended after reaching the peak displacement and there was still residual displacement after the explosion. At this time, both sides of the vault were damaged. The 12 mm and 16mm diameter BBCAs descended rapidly after reaching the peak displacement and finally returned to zero. Thus, the arch structure still maintained elastic deformation. By increasing the amount of TNT to 0.5 kg, the arch was significantly damaged and a large area of the concrete was broken at the top of the vault and obvious cracks appeared at the hance. At this time, the three BBCAs showed different behaviors after reaching the peak displacement. The displacement of the 8mm BBCA continued to increase and reached the maximum of 1.92 mm and then dropped. The maximum displacement of the 12 mm BBCA reached 1.31 mm and then decreased. The maximum displacement of the 16 mm BBCA reached 1.29 mm and the residual displacement of the vault was less than those of the 8 mm and 12 mm BBCAs.

From the above analysis, the contribution of the BFRP bars was not significant when the arch was in the elastic deformation. While the arch structure was severely damaged, the BFRP bars could make a significant contribution to the blast resistance of the arch. The larger the diameter of the BFRP bars was, the greater the contribution to the elastic deformation capacity of the arch structure was.

### 4.4. Acceleration of the Vault

**Figure 13:**
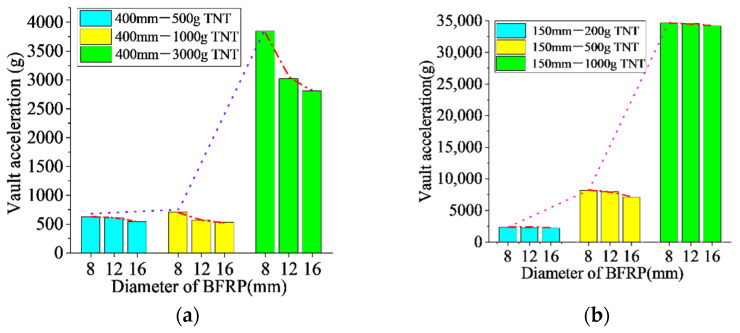
Peak acceleration at vault under various anti-explosion conditions: (**a**) standoff distance of 0.4 m, (**b**) standoff distance of 0.15 m.

Due to a large fluctuation in the time history curve of the vault acceleration, only the peak values were analyzed. Figure 13 shows the comparison of peak acceleration under various anti-explosion conditions. 

As shown in Figure 13, an increase in the diameter of the BFRP bars could reduce the peak acceleration at the arch vault, but the effect was not so significant. When the standoff distance was the same, and as the amount of TNT increased, the peak acceleration at the vault also increased significantly. With the standoff distance of 0.4 m, when the amount of TNT was increased from 1 kg to 3 kg, the peak acceleration at the vault increased from 597.4 g (the average peak value under the conditions of D, E, and F) to 3224.5 g (the average peak value under the conditions of G, H, and I) with an increase of 439%. With the standoff distance of 0.15 m, when the amount of TNT was increased from 0.5 kg to 1 kg, the average peak value under the conditions of M, N, and O increased from 7757.9 g to 34,474.3 g, with an increase of 344%. When the standoff distance was fixed, the influence of the amount of TNT on the peak acceleration was significant.

### 4.5. Strain of the BFRP Bars

The use of BFRP bars has many advantages. For example, the elastic modulus is much closer to that of concrete compared with steel bars and shows obvious failure characteristics before the failure. In this paper, the strain curves of the BFRP bars on the inner side of the vault and the hance were selected for the analysis, because the outer BFRP bars of the vault were always compressed while the inner BFRP bars were more subjected to the tensile stress. The bars mainly bear the tensile stress, so it was more meaningful to study the inner bars.

**Figure 14:**
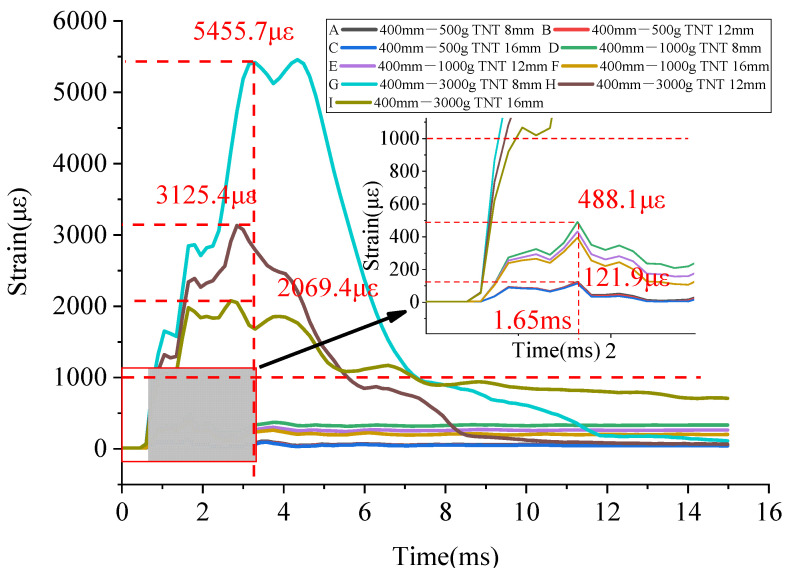
Time history curves of the strain of the BFRP bars at the vault under various working conditions with a standoff distance of 0.4 m.

With the standoff distance of 0.4 m, the strain of the BFRP bars on the vault with different diameters is shown in Figure 14.

When the scaled distance was varied, the strain of the BFRP bars reached its first peak point at 1.65 ms. However, when the amount of TNT was 3 kg, the BFRP bars had other strain peak points after reaching the first peak point.

Since integral modeling was adopted and the difference in the bonding performance between the BFRP bars and the concrete was not considered, the deformation of the concrete was identical to that of the BFRP bars. That is, the strain of the BFRP bars was equal to that of the concrete. According to Figure 14, when the standoff distance was 0.4 m and the amount of TNT was 0.5 kg and 1 kg, the peak strain of the BFRP bars was less than 1000 με indicating that the concrete arch was still in the elastic deformation. The change in the diameter of the BFRP bars had little effect on the peak strain. When the amount of TNT was increased to 3 kg, the peak strain of the BFRP bars exceeded 1000 με, and the BFRP bars bore most of the tensile stress. The peak strain could be significantly reduced by changing the diameter of the BFRP bars. As shown in Figure 14, when the amount of TNT was 3 kg, an increase in the diameter of the BFRP bars significantly reduced the peak strain from 5455.7 με (the BFRP bars with a diameter of 8 mm) to 3125.4 με (the BFRP bars with a diameter of 12 mm), with a decrease of 42.7%. When the diameter of the BFRP bars was 16 mm, the peak strain was reduced to 2069.4 με with a decrease of 62.1% compared with that of the BFRP bars with a diameter of 8 mm.

As shown in Table 11, with the same standoff distance, the peak strain increased with an increase in the amount of TNT (such as A, D, G or J, M, P) and decreased with an increase in the diameter of the BFRP bars (such as A, B, C or J, K, L). With the same amount of TNT, the peak strain increased as the standoff distance decreased. However, when the standoff distance and the amount of TNT were both different, the peak strain could not be compared. With the same standoff distance or the amount of TNT, the damage degree could be judged preliminarily by comparing the scaled distance. However, the use of the scaled distance to determine the structural damage had some limitations.

**Figure 15:**
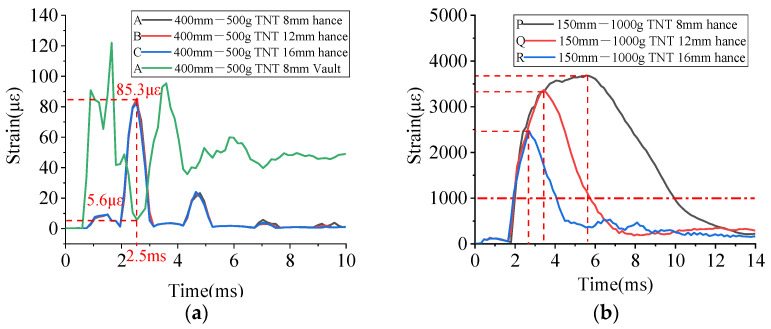
Time history strain curves of the BFRP bars at the vault and the hance under the working conditions of: (**a**) A, B, C and (**b**) P, Q, R.

In Figure 15a, the time history curves of the strain of the BFRP bars under the working conditions of A, B, and C were selected for comparison. It can be seen that the strains at the vault and the hance were ‘alternating’. At the beginning of the explosion, the vault was directly stressed and the strain of the BFRP bars quickly reached the peak value. Then the blast wave was transmitted from the vault to the hance. The strain of the BFRP bars on the hance began to increase and reached the peak value at the moment when the strain of the vault reached the minimum value after having the peak value.

As shown in Figure 15b, the peak strain of the BFRP bars at the hance under the working conditions of P, Q, and R far exceeded the tensile strain of ordinary concrete, and the BBCA was damaged. An increase in the diameter of the BFRP bars could significantly reduce the peak strain. BFRP bars played an increasingly important role in the blast resistance after the BBCA was damaged.

## 5. Conclusions

In this paper, the dynamic responses of BBCA structures under various blast conditions were studied through numerical simulation, and the macroscopic failure, pressure, displacement, acceleration, and strain were analyzed. The key factors affecting its anti-blast performance were discovered and the feasibility of using BFRP bars in a concrete arch was verified. The main conclusions are as follows:

- The numerical model proposed in this paper could analyze the dynamic response of the shallow-buried arch structure under the blast load, according to the comparison of the macroscopic damage and data between the tested model and numerical model. The BFRP bars could improve the dynamic bearing capacity of the reinforced concrete arch structure in protective engineering.
- The damage of exploded BBCA was divided into five levels: no damage, slight damage, obvious damage, severe damage, and collapse. BFRP bars could effectively mitigate the damage degree of shallow-buried underground protective arch structures under the explosive loads.
- A change in the diameter of the BFRP bars had a slight effect on the dynamic pressure on the vault. Theoretical analysis results indicate that the pressure on the vault is determined by the explosion load and the reflected pressure coefficient during an explosion. Through the calculation of the pressure on the vault, the damage to the structure can be predicted.
- The relationship between the displacement of the vault and the diameter of BFRP bars is affected by the scaled distance. When the scaled distance is small, the vault displacement changed slightly with a change in the diameter of the BFRP bars. However, the vault displacement decreased markedly with an increase in the diameter of the BFRP bars, when the scaled distance is large.
- Increasing the diameter of the BFRP bars could reduce the peak acceleration of the arch vault, but the effect was not so significant. Therefore, when using BFRP bars to reinforce the structure, it seems this can be used as an auxiliary measure to achieve the effect of vibration reduction.
- When the concrete cracks and the arch structure undergoes plastic deformation, the high-strength BFRP bars could significantly help the arch concrete to maintain explosion resistance and mechanical performance.

## Figures and Tables

**Table 1:** Parameters of the TNT material model and the equation of state.

Parameters	Definition	Value
ρ	Density of the TNT material	1630 kg/m^3^
D	Detonation velocity	6.718 m/s
PCJ	Chapman–Jouget pressure	18,500 Pa
E0	Detonation energy per unit volume and initial value for E	8 × 10^9^
V0	Initial relative volume, which gives the initial value for V	1

**Table 2:** Parameters of the air material model and the equation of state.

Parameters	Definition	Value
ρ	Density of the air material.	1293 kg/m^3^
PC	Pressure cutoff	0
MU	Dynamic viscosity μ	0
TEROD	Relative volume. $\frac{V}{V0^{\prime }}$, for erosion in tension	0
CEROD	Relative volume, $\frac{V}{V0^{\prime }}$, for erosion in compression	0
C1,C2,C3...C9	The 0,1,2,3...9th polynomial equation coefficients, which are zero when the state equation is used to simulate air	0
E0	Initial internal energy per unit reference volume	2.53 × 10^5^ J/m^3^
V0	Initial relative volume	1

**Table 3:** Parameters of the soil material model.

Parameters	Definition	Value
ρ	Density of the soil material.	1810 kg/m^3^
G	Shear modulus	6.39 × 10^7^ Pa
KUN	Bulk modulus for unloading used for VCR = 0	3 × 10^10^ Pa
PC	Pressure cutoff for tensile fracture (<0)	−6900 Pa
VCR	Volumetric crushing option	0
REF	Use reference geometry to initialize the pressure	0

**Table 4:** Parameters of the concrete material model.

Parameters	Definition	Value
ρ	Density of the concrete material	2400 kg/m^3^
IRATE	Rate effects options: EQUATION 1: Rate effects model turned on	1
FPC	Unconfined compression strength, f ‘C	3.38 × 10^7^ Pa
DAGG	Maximum aggregate size. If left blank, default for DAGG is 19 mm	0

**Table 5:** Parameters of the BFRP bars material model.

Parameters	Definition	Value
ρ	Density of the bars material	2050 kg/m^3^
E	Young’s modulus	4.55 × 10^10^ Pa
PR	Poisson’s ratio	0.3
SIGY	Yield stress	1.031 × 10^9^ Pa
ETAN	Tangent modulus	0
FS	Effective plastic strain for eroding elements	0.023

**Table 6:** Multi-condition parameters.

Standoff (m)	TNT (kg)	Scaled Distance (m/kg^1/3^)	Diameter of BFRP Bars (mm)	Group
0.4	0.5	0.504	8	A
			12	B
			16	C
	1	0.4	8	D
			12	E
			16	F
	3	0.277	8	G
			12	H
			16	I
0.15	0.2	0.256	8	J
			12	K
			16	L
	0.5	0.189	8	M
			12	N
			16	O
	1	0.15	8	P

**Table 7:** Mechanical parameters of the materials.

	Density (kg/m^3^)	Elastic Modulus (GPa)	Yield Strength (MPa)	Elongation (%)
**Concrete**	2400	33.8	33.8	-
**BFRP Bars**	2050	45.5	1031	2.55

**Table 8:** Comparison of the peak pressure and acceleration values of experiments and simulation.

	Experiments	Simulation	Difference
Peak pressure (MPa)	20.7	27.3	24%
Peak acceleration (g)	1758.5	2197.4	20%

**Table 9:** Pressure on the arch vault.

Group Name	A	D	G	J	M	P
$P_{0}$	3.06	7.28	21.47	26.9	27.3	50.5
$P_{1}$	1.27	2.40	6.60	8.20	18.88	35.65
$P_{0}/P_{1}$	2.41	3.03	3.25	3.28	1.45	1.42

**Table 10:** Impulses under various working conditions.

Group Name	A	D	G	J	M	P
Impulse(N·S)	2.73	7.51	24.18	11.66	13.64	45.61

**Table 11:** Peak strain of the BFRP bars on the vault under various working conditions.

**Group name**	A	B	C	D	E	F
**Peak strain (** με **)**	121.9	117.3	110.8	488.1	429.3	391.9
**Group name**	G	H	I	J	K	L
**Peak strain (** με **)**	5455.7	3125.4	2069.4	1524.69	773.555	639.032
**Group name**	M	N	O	P	Q	R
**Peak strain (** με **)**	4857.8	3110.17	1982.38	5623.04	3373.85	3173.66

## Data Availability

The data presented in this study are available on request from the corresponding author.
